# Elephantiasis Arabum

**Published:** 1870-01

**Authors:** 


### Elephantiasis Arabum.

Among the more positive practical advances of recent date may be mentioned the fact, now so well established, of the marked influence exerted upon a limb affected by elephantiasis arabum brought about by arresting the circulation through the main artery of the part. Many successful cases have been reported during the last few years. Ligature of the artery has been usually resorted to, but that this is not always necessary, is proved by Vanzett's case, where a cure was effected by digital compression alone.

The last summary on this subject is given by Dr. Fisher, of Hanover, who tabulates the cases which had occurred up to date, twenty-one in number. Out of these, eleven are reported cured, nine entirely, and two very nearly. In two cases there was improvement. In seven no improvement or death was the result; and one, Vanzetti's case, was cured by digital compression. The table proves that this method, although not necessarely successful, is yet an improvement on the treatment previously in use. The idea of curing elephantiasis by interrupting the circulation of a limb originated, it appears, with Dr. Dufour, since, in an article written by him in the *Gazette Hebd.*, 1863, he states that his first attempts in this direction date back thirty years; in other words, he was already experimenting the idea in 1833.—*N. Y. Med. Journal.*

